# Molar Macrowear Reveals Neanderthal Eco-Geographic Dietary Variation

**DOI:** 10.1371/journal.pone.0014769

**Published:** 2011-03-18

**Authors:** Luca Fiorenza, Stefano Benazzi, Jeremy Tausch, Ottmar Kullmer, Timothy G. Bromage, Friedemann Schrenk

**Affiliations:** 1 Department of Palaeoanthropology and Messel Research, Senckenberg Research Institute, Frankfurt am Main, Germany; 2 Palaeoanthropology Department, Faculty of Arts and Science, University of New England, Armidale, Australia; 3 Department of Anthropology, University of Vienna, Vienna, Austria; 4 Department of Science, Borough of Manhattan Community College, New York, New York, United States of America; 5 Institute of Ecology, Evolution & Diversity, Johann Wolfgang Goethe University, Frankfurt am Main, Germany; 6 Departments of Biomaterials and Biomimetics and Basic Science and Craniofacial Biology, New York University College of Dentistry, New York, New York, United States of America; University of Delaware, United States of America

## Abstract

Neanderthal diets are reported to be based mainly on the consumption of large and medium sized herbivores, while the exploitation of other food types including plants has also been demonstrated. Though some studies conclude that early *Homo sapiens* were active hunters, the analyses of faunal assemblages, stone tool technologies and stable isotopic studies indicate that they exploited broader dietary resources than Neanderthals. Whereas previous studies assume taxon-specific dietary specializations, we suggest here that the diet of both Neanderthals and early *Homo sapiens* is determined by ecological conditions. We analyzed molar wear patterns using occlusal fingerprint analysis derived from optical 3D topometry. Molar macrowear accumulates during the lifespan of an individual and thus reflects diet over long periods. Neanderthal and early *Homo sapiens* maxillary molar macrowear indicates strong eco-geographic dietary variation independent of taxonomic affinities. Based on comparisons with modern hunter-gatherer populations with known diets, Neanderthals as well as early *Homo sapiens* show high dietary variability in Mediterranean evergreen habitats but a more restricted diet in upper latitude steppe/coniferous forest environments, suggesting a significant consumption of high protein meat resources.

## Introduction

### Neanderthal diet

The study of dietary habits together with paleoecological analyses, allow researchers to obtain information regarding subsistence strategies in ancient human populations. In particular, many scientists have attempted to reconstruct the Neanderthal diet using associated faunal remains and lithic industry [1]–[3], stable isotope signatures [4]–[10] and dental microwear analysis [11]. These techniques suggest that Neanderthals from northern and middle latitudes were primarily active hunters subsisting on large and medium sized herbivores, while the southern Neanderthals from Mediterranean coastlines had a more diversified diet enriched by the exploitation of small animals and marine shellfish [3], [12]–[15]. Although a few studies indicate that early *Homo sapiens* were also active hunters focusing mainly on the consumption of terrestrial herbivores [9], [16], the analysis of faunal assemblages, stone tool technologies and stable isotopic studies indicate that early *Homo sapiens* exploited a broader dietary spectrum than Neanderthals [3], [12], [17]–[21]. Based on these data, Hockett and Haws [3] suggested that such a restricted diet in Neanderthals was lacking in essential nutrients, which could have increased maternal and fetal-to-infant mortality rates while also decreasing overall life expectancy. However, along Mediterranean coastlines where climatic fluctuations were less severe, isolated glacial refuges possibly existed. These likely would have contained a wide variety of dietary resources that may have enriched the Neanderthal diet and prolonged their survival [14]. Alternatively, a more diversified early *Homo sapiens* diet could have led to a demographic expansion resulting in increased competition with Neanderthal populations [2], [3].

New archaeological and paleontological evidence suggests that Neanderthals probably did, to some extent, exploit broader, more diverse food types. The Middle Paleolithic Mousterian cave deposits of Amud and Kebara (Israel) contain numerous plant fossils such as legumes, pistachios and acorns [22]–[24]. Also, microscopic examination of stone tools found at the Mousterian site of La Quina (France) show evidence of plant processing [25]. Finally, the existence of microfossil plant remains trapped in the dental calculus of Shanidar 3 of northern Iraq also may indicate the dietary intake of plant foods [26].

High Neanderthal dietary variation has also been suggested from dental microwear studies [27], [28]. The analysis of buccal microwear has shown that widely distributed Neanderthals from Europe and the near East of the Middle Paleolithic were characterized by heterogeneous microwear patterns. This heterogeny is inferred to have been caused by the exploitation of varied food resources reflecting climatic fluctuations rather than geographic dispersion [27]. Alternatively, a recent study of occlusal microwear indicates strong eco-geographic dietary variation in Neanderthals while also demonstrating the exploitation of a large dietary spectrum [28].

### Tooth wear

Normal (non-malocclusal) tooth wear is a dynamic, necessary and natural process caused by two main factors: attrition and abrasion [29]–[32]. Attrition is defined as the mechanical wear produced by the contacting surfaces of opposing teeth. Attritional wear produces well-defined highly pitched, flat surfaces called wear facets [31]–[33]. Wear facets are generally created by contact during normal mastication [34]–[36]. However, wear facets can also be produced by other non-masticatory activities such as bruxism (a pathological grinding of the teeth) [33]. Abrasion is produced by the friction of exogenous materials forced over the tooth surfaces [29]–[31]. Some foods themselves are abrasive (seed husks etc.). However, other foreign objects can also cause abrasion such as dust present in the environment or elements accidentally introduced with food preparation [29], [31]. Abrasion can also be due to tools used in oral hygiene [30], [37]. The action of food on a tooth surface is not anatomically specific; it does not create distinct and localized wear rather occurring over the whole occlusal surface [31].

The masticatory cycle begins with vertical mandibular movements (puncture-crushing), during which time the food is pulped and tooth-to-tooth contact is rare [38]–[41]. This is followed by a rhythmic chewing phase, or power stroke, wherein the attritional contacts between opposing teeth produces wear facets [38]–[41]. The power stroke is divided into two phases: phase I occurs when opposing molar crests shear past one another until the food is crushed between basins and cusps upon reaching centric occlusion; phase II consists of an anterior-medial movement of the lower molars on the chewing side until they move out of centric occlusion and the food is processed by grinding. The chewing cycle terminates with jaw opening [38]–[41].

The mastication of food items having different physical properties, such as abrasiveness, toughness, hardness and brittleness, requires different masticatory processing [38]–[43]. Raw meat is a tough, fibrous foodstuff with low-abrasiveness [28], [44]–[45]. The texture of plant materials is extremely variable even within species and different anatomical parts of plants can vary in physical properties. Buds, flowers and shoots are usually soft while the veins and stems of leaves, seeds and roots represent a tougher material [46]. Additionally, many plants contain silica phytoliths which renders them highly abrasive as a food type. In order to mechanically break down meat and leaves, masticatory shearing is required.

Tooth size and shape reflect the selective, functional pressures of ingested food physical properties [44]. The varied physical properties of different food types require distinct tooth morphologies to break them down [47]. While the shape of unworn teeth can suggest what a tooth is capable of processing, functional tooth wear can tell us what a tooth was actually used for [48]. Tooth wear is therefore the result of the complex interactions between attrition and abrasion, each with a varying density and duration thus generating a multitude of different wear patterns [31]. Consequently, tooth wear studies in prehistoric populations reveal information about diet, age at death, demography, life history, tooth association, food processing methods and other cultural and para-masticatory activities [36], [49]–[53].

### Dental occlusal compass

The occlusal fingerprint analysis (OFA) method is based on the comparison of phase I and phase II wear facet areas, dip and dip directions measurements, analyzing individual occlusal compasses [54] which describe the major movements of a tooth antagonistic cusps and basins through three-dimensional space [43]. The OFA method provides information about development, spatial position and enlargement of wear facets [54]–[55]. Jaw movements and the resulting occlusal contacts are highly correlated. This means that the information obtained from occlusal compass analysis can be utilized to understand how wear facets are formed and what movements are responsible for occlusal surface contacts [43], [54], [56]–[59]. Molar macrowear, as opposed to dental microwear, is a cumulative process which occurs during the individuals' lifetime and thus reflects long term diet [60].

Since there is a close relationship between jaw movements, occlusal wear and a food's physical properties [38]–[43], [60], interpretations of masticatory behavior from wear facet analyses allow us to reconstruct the dietary habits of fossil or extant species in which tooth to tooth occlusion occurs. The proportion of crushing and shearing wear in mammals has already been extensively used for dietary interpretations [39], [60]–[64]. The OFA method itself has so far been used to reconstruct the diets of *Australopithecus africanus*, *Australopithecus afarensis* and *Paranthropus robustus* [59] and it has also been employed in an experimental study which indicated a specific wear pattern associated with a daily vegetarian diet in humans [54]. Moreover, the OFA method has been recently used to analyze the relationship between dental morphology and tooth wear pattern in maxillary first molars of Neanderthals and *Homo sapiens* [65].

In this study, we analyze the maxillary molar macrowear of Neanderthals and early *Homo sapiens*, applying the occlusal fingerprint analysis method (OFA) to explore the relationship between eco-geographic variation and dietary mode. In particular, we examine whether Neanderthal diets consisted almost solely of animal proteins and whether their wear pattern is similar to those of the ‘carnivorous’ hunter-gatherers included in this study (Inuit, Vancouver islanders and Fuegians). Alternatively, a wear pattern different from those of the ‘carnivorous’ hunter-gatherers will indicate the exploitation by Neanderthals of broader dietary resources.

Moreover, if Neanderthals and early *Homo sapiens* had two different dietary strategies, as suggested from previous analyses, occlusal wear pattern should be taxonomic-dependent. If, instead, this hypothesis is not verified, similarities in wear pattern will indicate the exploitation of similar food resources.

## Results

### Phase I and phase II facet distribution in modern hunter-gatherers

**Figure 1:**
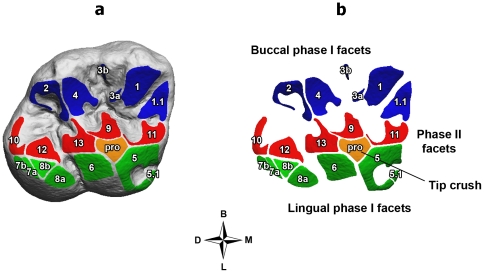
Occlusal wear pattern of first maxillary molars in Neanderthals. 3D polygonal tooth model **(a)** and wear facets virtually detached **(b)**, divided into buccal phase I facets (blue), lingual phase I facets (green), phase II facets (red) and tip crush areas (orange). Wear facet numbering system (1–13) after Kullmer et al. [52]. The division of homologous wear facets into more parts (as facet 3) is shown by the letters *a* and *b*. Orientation: buccal (B); distal (D); mesial (M); lingual (L).

In the Inuit, Vancouver islander and Fuegian groups, buccal phase I facets (Fig. 1) represent more than 47% of the entire occlusal wear area, while in the Khoe-San and the Australian aboriginal sample buccal phase I facets are 45% and 46.7%, respectively (Table 1). Alternatively, Inuit, Vancouver islanders, and Fuegians are characterized by lingual phase I facets with values lower than 17% of total occlusal area while in the Khoe-San and Australian aborigines, lingual facets are higher than 20%.

**Table 1:** Descriptive statistics of relative facets area within the modern hunter-gatherer groups.

		Buccal phase I		Lingual phase I		Phase II	
Group^*^	N	Mean	SD	Mean	SD	Mean	SD
Inuit	10	49.7	4.1	15.5	5.0	33.4	5.4
Vancouver is.	15	47.3	3.4	16.9	9.5	32.6	9.5
Fuegians	7	47.1	2.0	13.9	6.3	30.7	8.5
Australians	3	46.7	1.1	21.3	4.5	30.0	6.2
Khoe-San	7	45.0	4.6	27.1	5.5	24.4	4.4

The proportion of phase II facets are similar in all the modern hunter-gatherer groups (between 30% and 33%) with the exception of the Khoe-San in which phase II areas are less than 25%. The Vancouver islanders show the highest variation, especially in lingual phase I facets and phase II facets (standard deviation  = 9.5). The modern hunter-gatherer group shows a linear correlation between lingual phase I facets and phase II facets (*r* = −0.7788, *p*<0.001) showing that as phase II facets increase, the relative areas of the lingual facets decrease.

**Figure 2:**
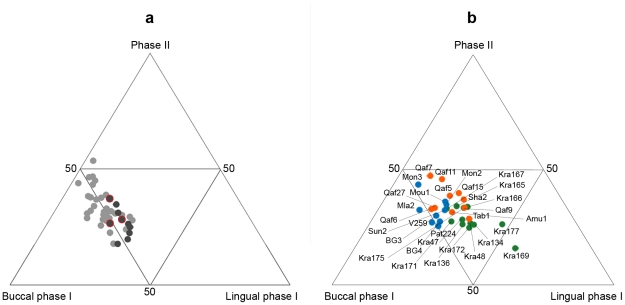
Ternary diagrams of relative wear facet areas. Wear facet distribution in (a) the modern hunter gatherer sample and (b) fossil specimens. The ternary diagram graphically depicts the proportions (%) of three variables (in this case: buccal phase I, lingual phase I and phase II relative wear facet areas) as positions in an equilateral triangle. Each base of the triangle represents a proportion of 0%, while the vertices correspond to a proportion of 100%. The modern sample is divided in predominantly meat-eating (Inuit, Vancouver islanders and Fuegians; grey) and mixed-diet hunter-gatherers (Khoe-San and Australian aborigines; black). There is a strong linear correlation between lingual phase I facets and phase II facets (*r* = −0.7788, *p*<0.00). The ‘carnivorous’ group is characterized by large buccal phase I facets, large phase II facets and less-developed lingual phase I facets. Alternatively, the mixed-diet sample shows large lingual phase I facets, small phase II facets and less-developed buccal phase I facets. The Australian aborigine specimens (circled red) tend to show overlap with the meat-eating and Khoe-San hunter-gatherers. Fossil specimens have been grouped in eco-geographical contexts: deciduous woodland (DEW, in green), Mediterranean evergreen (MED, in orange) and steppe/coniferous forest (SCF, in blue). The DEW group shows discrete variability and large lingual phase I facets. The SCF group resembles the meat-eating hunter-gatherers with large buccal and phase II facets. Finally, the MED group shows strong development of phase II facets. In particular, the Mediterranean Neanderthal specimens (Sha2, Tab1 and Amu1) tend to overlap with the DEW sample. The MED specimens Qafzeh 6 and 27 plot close to the SCF group. Fossil specimen labels: Amu  =  Amud; BG  =  Barma Grande; Kra  =  Krapina; Mla  =  Mladeč; Mon  =  Monsempron; Mou  =  Le Moustier; Pat  =  Abri Pataud; Qaf  =  Qafzeh; Sha  =  Shanidar; Sun  =  Sungir; Tab  =  Tabun; V  =  Vindija.

If all the three variables are considered, they can be graphically represented by means of the ternary plot (Fig. 2a). The ternary diagram shows the separation between predominantly meat-eating (Inuit, Vancouver islanders and Fuegians; in light grey) and mixed-diet hunter-gatherers (Khoe-San and Australian aborigines; in black), illustrating also the linear relation between lingual phase I facets and phase II facets. The one-way NPMANOVA test confirms the separation between meat-eating and mixed-diet hunter-gatherers, revealing significant differences between these two groups (*p* = 0.037, corrected with Bonferroni). However, the ternary diagram also illustrates high wear pattern variability and overlapping, which is mostly due to the Australian aborigine sample, which plots between meat-eaters and Khoe-San although they are closer to the latter. Statistically significant differences are found between Khoe-San and Inuit (*p* = 0.009), while the comparison between Khoe-San and Fuegians is close to the significance level (*p* = 0.056) (Table 2).

**Table 2:** Pairwise comparison between modern hunter-gatherer groups.

Group	Inuit	Vancouver is.	Fuegians	Australians	Khoe-San
Inuit	***				
Vancouver is.	1	***			
Fuegians	1	1	***		
Australians	1	1	1	***	
Khoe-San	**0.0090**	0.2730	0.056	1	***

One-way NPMANOVA based on relative areas (%) of buccal phase I facets, lingual phase I facets and phase II facets. Corrected p-values (Bonferroni), significant p-values (<0.05) are highlighted in bold.

### Phase I and phase II facet distribution in Neanderthals and early Homo sapiens

When relative facet areas of Neanderthals are compared with early *Homo sapiens* specimens belonging to the same environment, no significant differences are found (NPMANOVA: for the steppe and coniferous forest group *p* = 0.3149; for the Mediterranean group *p* = 0.0812). Buccal phase I facet areas in the steppe and coniferous forest group reach 45% of the total occlusal wear area, followed by Mediterranean and deciduous woodland groups (39.3% and 36.5% respectively) (Table 3). Lingual phase I facets show values of 34.5% in the deciduous woodland group, 23.0% in the Mediterranean and 19.9% in the steppe and coniferous forest sample.

**Table 3:** Descriptive statistics of relative facets area (in %) within the fossil Biome groups.

		Buccal phase I		Lingual phase I		Phase II	
Group*	N	Mean	SD	Mean	SD	Mean	SD
DEW	12	36.5	5.2	34.5	9.1	27.8	5.3
MED	10	39.3	4.2	23.0	7.1	36.7	6.1
SCF	9	45.1	4.0	19.9	5.3	30.9	5.6

*Groups: Deciduous woodland (DEW), Mediterranean evergreen (MED) and Steppe/Coniferous forest (SCF).

The development of phase II facets is higher in the Mediterranean sample (36.7%), followed by the steppe and coniferous forest (30.9%) and the deciduous woodland groups (27.8%). The graphical representation obtained using the ternary plot demonstrates separation of the three groups, where the deciduous woodland group plots toward the lingual phase I corner, the steppe and coniferous forest specimens are distributed close to the buccal phase I corner and the Mediterranean group is positioned in the central part of the graph with some distribution toward the phase II corner (Fig. 2b). There is a slight overlap between the Mediterranean and deciduous woodland groups due to the Tabun 1 and Amud 1 specimens, while Qafzeh 6 and 27 (Mediterranean group) plot between the steppe and coniferous forest specimens. Results of one-way NPMANOVA show that the relative facet areas of all the groups are significantly different (Table 4).

**Table 4:** Pairwise comparison between fossil Biome groups.

Group*	DEW	MED	SCF
DEW	***		
MED	**0.0018**	***	
SCF	**0**	**0.0333**	***

*Groups: Deciduous woodland (DEW), Mediterranean evergreen (MED) and Steppe/Coniferous forest (SCF).

One-way NPMANOVA based on relative areas (%) of buccal phase I facets, lingual phase I facets and phase II facets. Corrected p-values (Bonferroni), significant p-values (<0.05) are highlighted in bold.

### Comparison between modern hunter-gatherers and biome-specific fossil groups

Relative areas of buccal, lingual and phase II facets are compared between modern hunter-gatherer and human fossil eco-geographical groups. Significant differences are obtained between Pleistocene hominins from deciduous woodland and meat-eating hunter-gatherers (NPMANOVA: for Inuit *p* = 0; for Vancouver islanders *p* = 0; for Fuegians *p* = 0.0015), while no differences are found between Pleistocene hominins from deciduous woodland and mixed-diet hunter-gatherers (Table 5). The Mediterranean group shows significant differences with Inuit (*p* = 0.03), Fuegians (*p* = 0.0045) and Khoe-San (*p* = 0.054), but not with Vancouver islanders and Australian aborigines. Finally, the steppe and coniferous forest group does not show any significant difference with any of the modern hunter-gatherer groups.

**Table 5:** Comparison between fossil and modern hunter-gatherer groups by biome.

Group*	Inuit	Vancouver is.	Fuegians	Australians	Khoe-San
DEW	**0**	**0**	**0.0015**	0.3855	0.3420
MED	**0.030**	0.1935	**0.0045**	1	**0.0054**
SCF	0.954	1	1	1	0.3420

*Groups: Deciduous woodland (DEW), Mediterranean evergreen (MED) and Steppe/Coniferous forest (SCF).

One-way NPMANOVA based on relative areas (%) of buccal phase I facets, lingual phase I facets and phase II facets. Corrected p-values (Bonferroni), significant p-values (<0.05) are highlighted in bold.

## Discussion

### Functional aspects of occlusal tooth wear in relation to diet

The analysis of modern hunter-gatherer occlusal wear pattern based on their relative areas, distinguishes the carnivorous populations from those dependent on a mixed diet.

We have discovered that buccal phase I wear facets are well-developed in the Inuit, Vancouver islander and Fuegian groups indicating a developed shearing capability. However, we find that these facets are slightly less pronounced in the Khoe-San and the Australian aborigines. Our results highlight that increased meat consumption in modern hunter-gatherers could be related to a proportionate increase in buccal phase I facets (shearing). As observed in numerous extant herbivores prominent buccal phase I facets occur in species consuming large amounts of tough, fibrous and flat food items [60]. The presence of large buccal phase I facets in non-human primates with diets rich in tough, fibrous foods [60] highlights the importance of these areas for processing pliant, tough food regardless of the taxonomic affiliation. Alternatively, Inuit, Vancouver islanders, and Fuegians are characterized by small lingual phase I facets which are prominent in the Khoe-San and Australian aborigines. The presence of large lingual phase I facets in these two groups indicates an increase in transverse mandibular movement, suggesting an increased reliance on hard, abrasive foodstuffs including roots, seeds, gums and other plant materials. This is in agreement with the observation [60] that herbivorous mammals with an omnivorous diet show more pronounced lingual phase I facets than those of obligate browsers and grazers. The proportion of phase II facets are similar in all the modern hunter-gatherer groups with the exception of the Khoe-San in which phase II areas are less developed. However, the phase II facets in the modern hunter-gatherer meat-eating group and Australian aborigines are more developed than those of the Khoe-San, which contradicts the proposal that increased phase II faceting is associated with a more frugivorous diet [60]. Since there is a significant correlation between an increase in phase II facets and decrease in lingual phase I areas, this change could be partly related to morphological constraints rather than feeding habits.

As seen in the ternary diagram (Fig. 2a), the modern hunter-gatherer groups tend to overlap along a continuum. This could be due not only to similar dietary habits but also to the use of comparable food processing techniques and/or cultural proclivities [49]. Thus, the use of food preparation methods (desiccation, smoking, cooking, freezing etc.) which introduce large amounts of foreign abrasive materials into the diet may have generated similar wear patterns.

A larger sample size with more specific occupational and dietary information for individual specimens would have yielded more accurate results. Despite that, the OFA method proved itself to be a useful tool in discriminating the diets of eco-geographically and/or temporally separate populations (e.g. there was a clear separation between meat-eating and mixed-diet hunter-gatherers). Moreover, the functional aspects of the results obtained here agree with those of previous works [39], [60]–[64], confirming the validity of the OFA method for detecting information about diet.

### Eco-geographical variation within the human fossil sample

Relative wear facet areas on Neanderthal and early *Homo sapiens* upper molars do not reflect any significant taxonomic differences, as is demonstrated for enamel thickness [66]. Instead, macrowear patterns reveal eco-geographical variation such that specimens belonging to either taxonomic group inhabiting similar environments show similar wear patterns.

As Neanderthal and early *Homo sapiens* maxillary molar morphology differs [67]–[70], the absence of wear pattern differences between them suggests that the variation in tooth morphology between these two groups is of little importance for wear facet development. This hypothesis is strengthened by a recent study where the development and enlargement of occlusal wear facets in Neanderthal and *Homo sapiens* first molars were shown to be independent of tooth cusp size [65]. This result is in agreement with previous findings where the analyses of species with similar diets but different dental morphologies showed comparable tooth wear [60].

The highly heterogeneous molar macrowear pattern of Neanderthals and early *Homo sapiens* specimens was also found in buccal [27] and occlusal [28] dental microwear studies, indicating the exploitation of diverse food materials.

Individuals occupying deciduous woodland environments show an occlusal wear pattern characterized by large lingual phase I facets and reduced buccal phase I facets. High variability in occlusal wear, as suggested by El Zaatari [28], could reflect the exploitation of highly diverse food resources (plants and animals) available in these types of environments. The reduction in buccal phase I facets is likely related to a decreased reliance on tough, fibrous items such as meat, while the increase of lingual phase I facets probably relates to an increased exploitation of hard, abrasive foods such as plant materials. The deciduous woodland group differs significantly from the Khoe-San hunter-gatherers, by having larger lingual phase I facets. This could indicate a larger proportion of plant materials in the diets of deciduous woodland individuals. This conclusion cannot be confirmed since fully vegetarian human populations could not be included in this study.

Neanderthals and early *Homo sapiens* from steppe and coniferous forest habitats show wear patterns similar to those of the predominantly meat-eating modern hunter-gatherers and Australian aborigines and only slightly different from the Khoe-San. The steppe and coniferous forest group is characterized by prominent buccal phase I facets, small lingual phase I facets, and phase II facet areas slightly larger when compared to those of deciduous woodland samples. They suggest a tooth wear pattern indicative of an increased reliance upon tough, fibrous foods such as meat. Although the meat-eating hunter-gatherers used in this study predominantly exploited marine food resources, it is not possible to distinguish between marine and terrestrial animal resources from dental wear studies [28].

Buccal microwear analysis indicates that Neanderthals from cooler OIS had a more abrasive diet than those from warmer OIS due to the exploitation of plant materials such as roots and tubers [27]. However, habitat deterioration during glacial periods resulted in a lowered diversity of edible plant foods in comparison to those present in warmer environments [28]. It is therefore unlikely that Neanderthals enduring cooler OIS exploited a broader dietary spectrum than those from warmer stages.

The occlusal wear pattern of the Mediterranean group is different from the predominantly meat eating modern hunter-gatherer and also from the Khoe-San hunter-gatherers. Within the Mediterranean sample, Neanderthal specimens Amud 1, Tabun 1 and Shanidar 2 show strong similarities with the deciduous woodland group, indicating the exploitation of varied food resources. Similarly, microwear signatures placed Amud 1 and Tabun 1 with the European Neanderthals inhabiting deciduous woodland habitats [28]. Additionally, numerous palaeobotanical remains of edible plants recovered from deposits at Amud [23] and Kebara [22], [24] and plant remains trapped in the dental calculus of Shanidar 3 [26], demonstrate that ‘near eastern’ Neanderthals consumed a wide variety of plant materials such as legumes, wild grasses, fruits and seeds. The Qafzeh specimens belonging to the Mediterranean group show a slightly different occlusal wear pattern characterized by the strong development of phase II facets. Although the functional significance of phase II facets is still debated [42], [60], [71]–[72], similarities between Australian aborigines and the Qafzeh specimens may suggest the exploitation of a broad dietary spectrum including animal proteins and plant foods. High wear pattern variability in Middle Paleolithic *Homo sapiens* from the Levantine region was also reported from dental microwear studies [28], [73]. In addition, a rich and diverse mammalian fauna discovered at Qafzeh, together with the presence of numerous marine shells collected and transported from the sea shore (the nearest archaic shoreline was about 45–50 km from the Qafzeh cave) [74], reveals a high level of foraging mobility and the ability to exploit varied food sources from neighboring environments, which may explain the elevated macrowear pattern variability found in the Qafzeh sample.

These results contradict those obtained by previous analyses where Neanderthals have been traditionally viewed as a species feeding mostly on animal proteins and more specifically large game animals. Methodological and conceptual limitations as well as geographic factors may have historically ‘conspired’ to produce narrow interpretations of Neanderthal diets. This historical bias appears to have caused many researchers to overlook the possibility that Neanderthals also exploited, to varying degrees, food resources other than meat. As bone fossilizes more readily than plants, the impression is often given that meat was the primary food resource [75]. For these reasons, the analysis of archaeological and paleontological remains tends to underestimate plant consumption and overestimate animal consumption [76].

Moreover, all stable isotopic studies of Neanderthal diets have been based only on specimens inhabiting the colder areas of north and central Europe [77]. Our analysis concurs with these studies of geographically restricted populations. However, we also suggest that Neanderthals from warmer environments may have exploited a much broader dietary resource base including a significant amount of plant materials.

Finally, the fossil samples high wear pattern variability could reflect the strong climatic fluctuations which occurred in Eurasia during the approximately 200,000 years of Neanderthal occupation. Consequently, large bio-geographical, chronological and climatic variations not only exposed Neanderthal populations to a wide variety of eco-geographical contexts, but also to substantial food resource diversity. Moreover, new isotopic studies and archaeological evidence suggests that Neanderthals [78] and early *Homo sapiens* [74] exploited large geographical ranges. A high mobility pattern suggests that they may have had access to various eco-geographical zones during their lifetime and, consequently, exploited many diverse food types.

Alternately, three of the modern hunter-gatherer populations included in this study inhabit environments characterized by low ecologic diversity but high productivity (Inuit = arctic climate, Vancouver islanders = temperate and cold maritime climate, Fuegians =  cold maritime and subantarctic climate). Thus, the wear ‘signal’ we are observing comes from ecologically restricted dietary choices. The remaining two populations inhabit areas with high seasonal ecological diversity but lower productivity (Khoe San =  semi-arid desert region, Australian aborigines =  semi-arid coastal areas). Here, the wear observed may have come from a seasonally restricted ‘signal’. And, importantly, these populations were sampled through a very brief geological timeframe (a few hundred years) during which climatic conditions were less variable than the fossil sample. These factors may, therefore, account for some of the wear pattern variation observed between the modern and fossil samples.

### Neanderthals and early Homo sapiens during OIS 3

The Neanderthal and early *Homo sapiens* specimens analyzed here, who inhabited the European continent during the cool OIS 3, show similar occlusal wear patterns resembling those of typical meat eating hunter-gatherer living in cold environments. This suggests that their diet was low in diversity consisting mainly of animal proteins.

If Neanderthals and early *Homo sapiens* from OIS 3 exploited different food sources, dietary variation should be reflected through the different use of teeth and their resultant wear patterns. However, the analysis of occlusal wear pattern in OIS 3 European Neanderthals and early *Homo sapiens* did not show any significant difference. The Neanderthal occlusal wear pattern indicates a diet based mostly on animal proteins, as suggested by previous studies. Likewise, the reduced variability in wear pattern and strong similarity with Neanderthals suggests that early *Homo sapiens* also exploited a restricted dietary spectrum dominated by the intake of animal proteins.

It is important, however to note that this result may be influenced by small sample size (Neanderthals, N = 4; early *Homo sapiens*, N = 5). Moreover, we cannot conclude that the two Pleistocene hominins competed directly for food resources during OIS 3 due to a lack of Neanderthal specimens chronologically contemporary to the early *Homo sapiens* sample. It will then be necessary to analyze a larger OIS 3 fossil sample, including also the latest Neanderthals (e.g. Saint-Césaire 1, Zafarraya or Vindija V-207 and V-208), to further test our results.

Despite small sample size, our results suggest that teeth are functionally related to eco-geographic dietary variation and that tooth wear patterns are independent of Neanderthal and early *Homo sapiens* taxonomic affinity, which is then a reflection of the intimate relationship that teeth have with the environment.

## Materials and Methods

### Sample data

**Figure 3:**
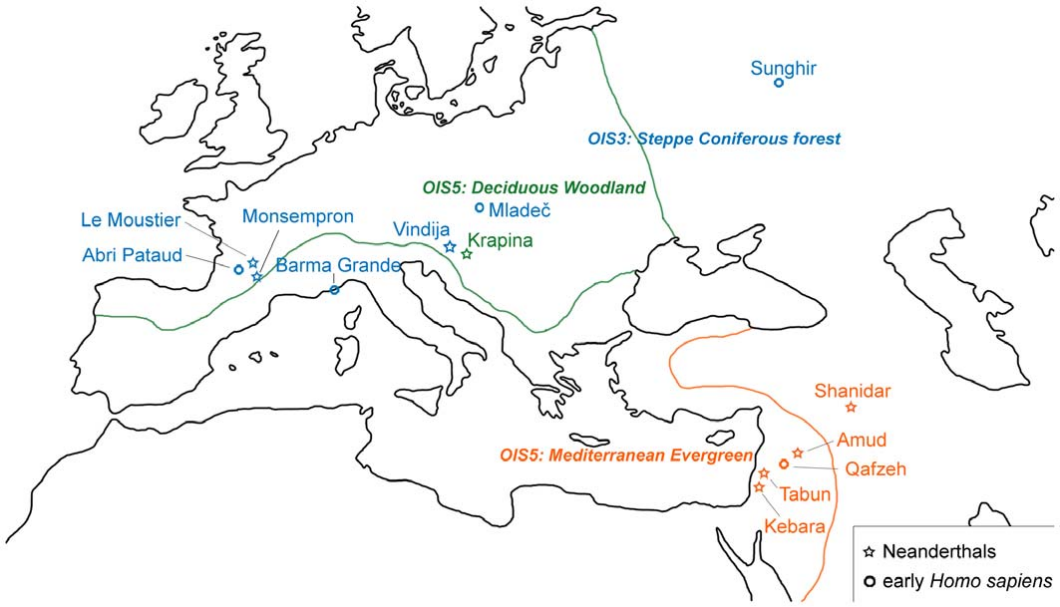
Neanderthals and EHS sites. The fossil sample analyzed in this study consists of 19 Neanderthals and 12 EHS. Fossil specimens have been grouped in eco-geographical contexts based on geographical area, approximate dating, and paleoenvironmental information derived from faunal analysis and palynology. Reconstruction of the vegetation is adapted from Van Andel & Tzedakis [75], who analyzed Oxygen Isotope Stages (OIS) between 150,000–25,000 years ago. Neanderthals and EHS are placed into three major eco-geographical colour coded categories: deciduous woodland (DEW, OIS 5, n = 12); Mediterranean evergreen (MED, OIS 5, n = 10) and steppe/coniferous forest (SCF, OIS 3, n = 9). (See also Table 6 and Supplementary Text).

The sample consists of 73 specimens, whereof 19 are Neanderthals, 12 are early *Homo sapiens* and 42 are modern and historic hunter-gatherers. Based on information regarding geographical area, approximate dating, together with paleoenvironmental information derived through faunal analysis, palynological data and pedology, the fossil specimens were grouped into eco-geographical categories. Vegetation reconstructions were based primarily on the work of Van Andel & Tzedakis [79], who described the European vegetation at different Oxygen Isotope Stages (OIS) between 150,000–25,000 years ago. Neanderthals and early *Homo sapiens* have been divided into three major eco-geographical groups which represent three different and specific environments: 1) deciduous woodland, 2) Mediterranean evergreen and 3) steppe/coniferous forest (Fig. 3, Table 6 and Text S1). The modern hunter-gatherer sample is composed of Inuit (n = 10), Vancouver islanders (n = 15), Fuegians (n = 7), Australian aborigines (n = 3) and Khoe-San (n = 7) (Table 6). Based on dietary information collated from the literature, Inuit, Vancouver islanders and Fuegians were grouped together as predominantly meat-eating hunter-gatherers [80]–[83], while Australian aborigines and Khoe-San were defined as mixed-diet hunter-gatherers [84]–[90].

**Table 6:** List of fossil and modern human specimens used.

Group	Individuals/population	Tooth	Wear	Locality	Dating (ka)	OIS	Biome	Diet
NEA (n = 19)	Krapina maxilla C 47	ULM1	2	Croatia	130±10	5e	DEW	
	Krapina maxilla D 48	ULM1	2	Croatia	130±10	5e	DEW	
	Krapina 134	URM1	2	Croatia	130±10	5e	DEW	
	Krapina 136	ULM1	2	Croatia	130±10	5e	DEW	
	Krapina 165	URM2	2	Croatia	130±10	5e	DEW	
	Krapina 166	URM1	2	Croatia	130±10	5e	DEW	
	Krapina 167	URM1	2	Croatia	130±10	5e	DEW	
	Krapina 169	URM2	2	Croatia	130±10	5e	DEW	
	Krapina 171	URM1	2	Croatia	130±10	5e	DEW	
	Krapina 172	URM2	3	Croatia	130±10	5e	DEW	
	Krapina 175	ULM2	2	Croatia	130±10	5e	DEW	
	Krapina 177	URM2	2	Croatia	130±10	5e	DEW	
	Tabun 1	ULM2	3	Israel	122±16	5e	MED	
	Monsempron 2	ULM1	3	France	100–120	5e	SCF	
	Monsempron 3	URM1	3	France	100–120	5e	SCF	
	Shanidar 2	ULM2	3	Iraq	?	?	MED	
	Amud 1	ULM2	3	Israel	55	3	MED	
	Vindija V259	ULM2	2	Croatia	42	3	SCF	
	Le Moustier 1	ULM1	2	France	41	3	SCF	
EHS (n = 12)	Qafzeh 5	ULM1	3	Israel	115±15	5d	MED	
	Qafzeh 6	ULM2	3	Israel	115±15	5d	MED	
	Qafzeh 7	ULM2	3	Israel	115±15	5d	MED	
	Qafzeh 9	ULM1	3	Israel	115±15	5d	MED	
	Qafzeh 11	ULM1	2	Israel	115±15	5d	MED	
	Qafzeh 15	URM1	2	Israel	115±15	5d	MED	
	Qafzeh 27	ULM1	3	Israel	115±15	5d	MED	
	Mladeč 2	ULM1	3	Czech Rep.	31	3	SCF	
	Barma Grande 3	ULM1	2	Italy	24,8	3	SCF	
	Barma Grande 4	ULM1	3	Italy	24,8	3	SCF	
	Sungir 2	URM1	2	Russia	26,2–27,2	3	SCF	
	Pataud 224	ULM1	3	France	21–22	2	SCF	
MHG (n = 42)	Inuit (n = 10)^1^^,^ 2			Greenland				Carnivorous
	Vancouver islanders (n = 15)^1^			Canada				Carnivorous
	Fuegians (n = 7)^1^^,^ 2			Argentina				Carnivorous
	Australian aborigines (n = 3)^2^			Australia				Mixed-diet
	Khoe-San (n = 7)^3^			South Africa				Mixed-diet

*Groups: Neanderthals (NEA), early *Homo sapiens* (EHS) and modern hunter-gatherers (MHG).

**Biomes: Deciduous woodland (DEW), Mediterranean evergreen (MED) and Steppe/Coniferous forest (SCF).

1 Natural History Museum of London.

2 Natural History Museum of Vienna.

3 University of Vienna.

**Figure 4:**
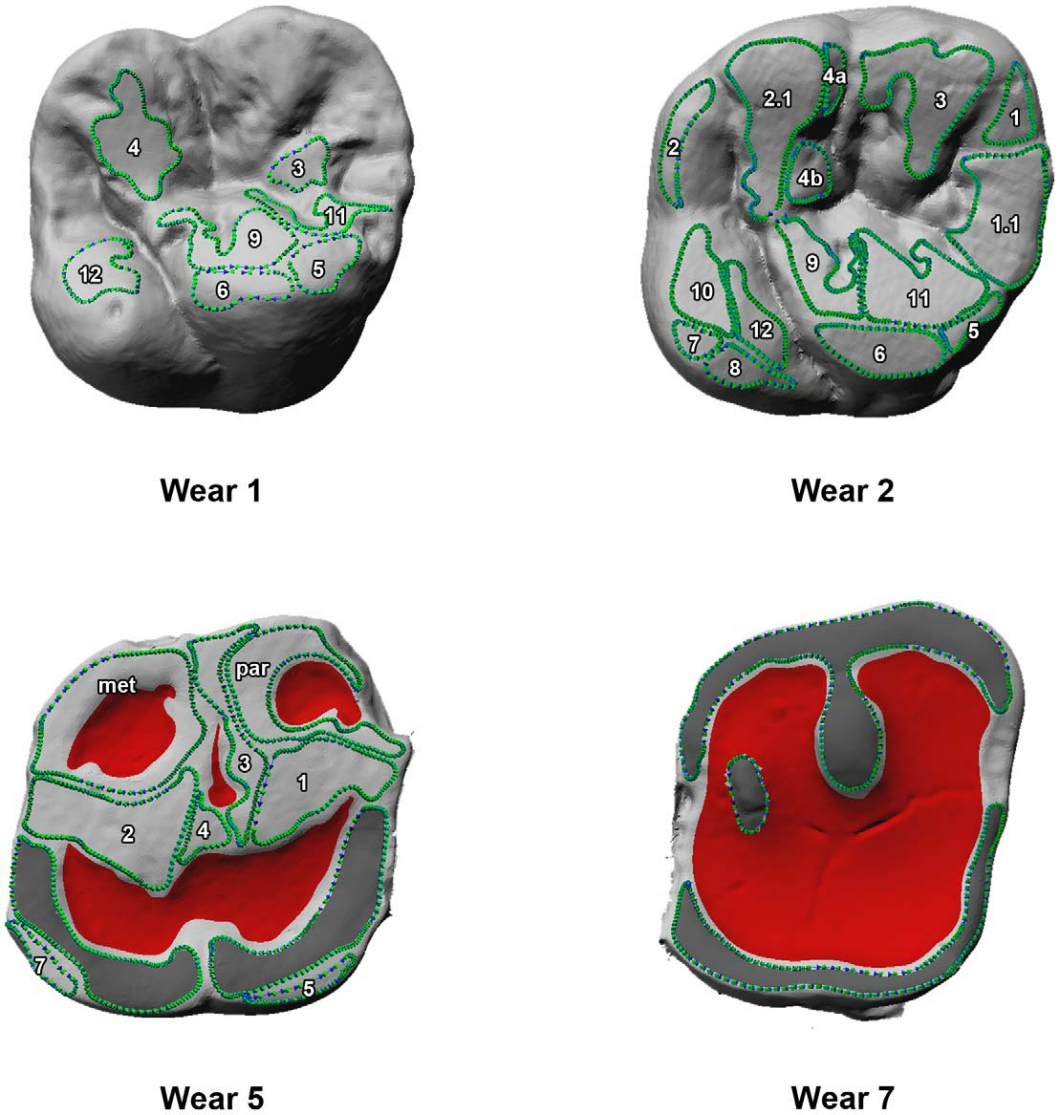
Dental wear stages. Wear stages (1, 2, 5 and 7) for maxillary first molars were determined by evaluating the amount of dentin exposure and cusp removal [50]. Wear facets are outlined in green, while dentinal areas are colored in red. The areas colored in dark grey (examples of wear stages 5 and 7) are the result of the coalescence of two (or more) wear facets which are no longer identifiable.

Lower molars were not considered as mandibles since were often absent from the sample specimens and, as such, sufficient molars were not available to form a robust lower molar sample set. Third molars are extremely variable [33], [91]–[92] and thus have been discarded. We have included only slightly worn first and second maxillary molars where facets were identifiable and did not coalesce. In heavily worn teeth, the occlusal surface is flattened and characterized by large dentinal areas. The wear facets tend to fuse and most of the information is consequently lost (Fig. 4).

Wear stage was determined by evaluating the amount of dentin exposure and cusp removal [50]. In order to create a more homogenous sample, we selected from each specimen only one maxillary molar in wear stage 2 (moderate cusp removal, with one or two pinpoint dentin exposures) and wear stage 3 (full cusp removal and some dentin exposure).

### Occlusal fingerprint analysis (OFA)

Wear facets were defined on the occlusal tooth surface following the terminology of Maier & Schneck [93], which identified a maximum 13 pairs of facets in hominoid molars (Fig. 1). Once wear facets were identified, they were grouped by chewing cycle phases [39], [60], defining three groups: lingual phase I facets (1, 2, 3 and 4), buccal phase I facets (5, 6, 7 and 8) and phase II facets (9, 10, 11, 12 and 13). We also recognized flat, worn areas on the tips of the four main upper molar cusps. These features have been named “*tip crush areas*” and show a circular shape without dominant directions of striations. They are believed to be produced during puncture-crushing where the maxillary cusps occlude with the basins of the mandibular molars [60], [94]. However, due to their small areas and to their low frequency (less than 10%), tip crush areas were not be further considered in the quantitative analysis.

For the study of occlusal wear patterns we applied the OFA method [54]–[55], using three-dimensional digital models originated from the surface scanning of dental casts. Original dentitions and high resolution dental replicas were moulded using a light viscosity polyvinylsiloxane silicone, Provil® Novo Light C.D.2 (Heraeus Kulzer GmbH). The negative replicas were then produced using a special gypsum (Everest® Rock, KaVo), possessing non-reflective properties and optimized for light scanning [95]. Three-dimensional digital models were generated using a white-light scanning system with a resolution of 55 µm (smartSCAN 3D, Breuckmann GmbH). Collection and alignment of the scan-data point clouds was carried out using optoCAT software (Breuckmann, GmbH). The 3D virtual models were further post-processed using PolyWorks® 10.1 (InnovMetric Software Inc.), a 3D model editing software package.

In order to ensure common initial specimen alignment, in the IMEdit module of PolyWorks® the cervical margin was manually delimited using the polyline tool [59], [96]. A marginal area of 0.2 mm above and below the cervical polyline was defined and a cervical plane was created and inserted by means of the least square, best fit method [59], [96]. To define each wear facet, a polyline was inserted manually on the digital model following the anatomical borders of the facet. Next, the triangles included within the facets perimeter were selected and the area in mm^2^ was automatically calculated. As larger teeth likely develop larger facets than smaller teeth (both displaying a similar degree of wear), a relative value was obtained dividing each facet area by the total wear occlusal area. The total wear occlusal area was calculated as the sum of the absolute area of each occlusal facet.

### Statistical analysis

Paleontological data are invariably incomplete [97]. Our fossil sample is rather heterogeneous chronologically and geographically such that it is only possible to identify small groups. An explorative data analysis for each variable (mean and standard deviation) for each group has been provided. As a small sample size prevents the assumption of a normal distribution, the statistical comparison necessarily was carried out using non-parametric tests. A Non-Parametric MANOVA (one-way NPMANOVA) analysis is a multivariate analogue to Fisher's *F*-ratio and is calculated directly from any symmetric distance or dissimilarity matrix [98]. Pairwise NPMANOVAs between all pairs of groups was based on three variables (relative areas (%) of buccal phase I facets, lingual phase I facets and phase II facets). The significance is computed by permutation of group membership (n = 10000) and results are provided using a post-hoc test (Bonferroni). Wear facet proportions were visualized using a ternary plot which represents the relative proportion of three variables which have to sum to 1 or 100%. Each variable is placed at the apex of a triangle and the sample is plotted as a set of points inside this triangle [97]. A sample consisting entirely of A will be placed at the corner ‘A’, while a sample consisting of equal proportions of A, B, and C will plot in the center [97]. The statistical analyses, including the ternary plots, were computed using the software PAST v.1.83 (PAlaeontological STatistics) [99].

## Supporting Information

Text S1 In this document each fossil specimen is placed in a determined eco-geographical context based on paleoenvironmental information derived through faunal analysis and palynological data. Moreover, detailed information (geography, climate, environment, diet and food processing methods) about the modern hunter-gatherer groups used in this study have been included.
(0.05 MB DOC)
