# Supplementary material for: Molar Macrowear Reveals Neanderthal Eco-Geographic Dietary Variation
Source: PLoS One. 2011 Mar 18;6(3):e14769. doi: 10.1371/journal.pone.0014769 (PMC3060801; doi:10.1371/journal.pone.0014769)
Supplement: Text S1 — In this document each fossil specimen is placed in a determined eco-geographical context based on paleoenvironmental information derived through faunal analysis and palynological data. Moreover, detailed information (geography, climate, environment, diet and food processing methods) about the modern hunter-gatherer groups used in this study have been included. (0.05 MB DOC) [file pone.0014769.s001.doc]

Supplementary Information

Supplementary Discussion

*Fossil sample*

The deciduous woodland sample (DEW) is composed only of Neanderthal specimens which occupied northern Croatia (Krapina) during OIS 5e [1]. This was a relatively warm period with vegetation comprised of deciduous forests and Mediterranean woodland [2].

The Mediterranean evergreen group (MED) is composed of Neanderthal specimens Amud 1, Tabun 1 and Shanidar 2 and by the early *Homo sapiens* specimens from Qafzeh. Amud 1 and Tabun 1 lived in the same region (Israel) at different times; OIS 3 and OIS 5e respectively [3,4]. Although the Levant was characterized by slightly different climatic conditions during these two periods [5, 6], it is reasonable to hypothesize a similar warm environment characterized by a vegetation consisting of shrubs and forested evergreen. Consequently, the two Neanderthals specimens are included in the same group. The dating of Shanidar 2 is dubious, incomplete and can only be placed generally between OIS 6 and OIS 4 [7]. However, faunal remains present at the site [8] indicate an open environment consisting of shrub vegetation which is similar to the Mediterranean zone and, therefore Shanidar 2 is considered as part of the Mediterranean group. The Qafzeh specimens occupied the Levant during the Middle Paleolithic OIS 5d [9]. The Qafzeh environment was characterized by hot, dry climate conditions [6] with a vegetation mainly consisting of Mediterranean evergreens [2].

Finally, the steppe/coniferous forest group (SCF) is composed of four Neanderthal specimens (Monsempron 2 and 3, Le Moustier 1 and Vindija V259) and five early *Homo sapiens* specimens (Mladeč 2, Barma Grande 3 and 4, Sungir 2 and Pataud 224). Although steppe and coniferous forest represent two different biomes, both occur in cold environments and may have both provided a low diversity of edible plants. Little dating information is available for the Monsempron sample. Although the beginning of Würm I has been posited and which corresponds to a warm climate [10], the presence of reindeer in the faunal assemblage and stone tool technology analysis suggests a colder climate. However, it likely corresponds to the end of OIS 4 or the beginning of OIS 3 [11] and, consequently it is possible to hypothesize steppe and/or coniferous forest type vegetation. Le Moustier of south-west France was occupied around 41 kya [12] which corresponds to a cold interval of OIS 3 [13]. This climate is confirmed by the presence of reindeer in the faunal assemblage [10]. The dating of specimen Vindija V259 of northern Croatia also corresponds to cold interval OIS 3 [14], and is therefore considered within the steppe/coniferous forest group. Mladeč 2 occupied central Moravia at the end of OIS 3 [15] where cold climatic conditions prevailed and confirmed by the faunal assemblage [16]. The Sungir specimen is also dated at the end of OIS 3 [17], a period in central Russia characterized by a cold, continental climate and typified by steppe and coniferous forest [18]. Barma Grande 3 and 4 occupied the Ligurian coastline of northern Italy between the transition from OIS 3 to OIS 2 [19] during which the biome is represented by (open environments and a reduction of coniferous forest [20]. Pataud 224 was found in south-western France and dated to 22 kya [21] which corresponds to OIS 2. The climate was dry and cold with the landscape dominated by coniferous forests [22].

*Modern hunter-gatherer sample*

The Khoe-San sample consists of seven specimens coming from the Kalahari and Cape Region collected by Rudolph Pöch during his trips to South Africa between 1907 and 1909 [23]. The Kalahari is a semi-arid, mostly flat, desertic region with an average altitude of about 1,000 m above sea level and lies within regions of Botswana, Namibia and South Africa. It is characterized by seasonal extremes in temperature and rainfall. Despite its general aridity, the northern and western regions experience higher annual precipitation and so the vegetation is more dense and less seasonal. Here, the vegetation is mostly composed of deciduous trees, shrubs and grasses adapted to erratic rainfall. During the rainy season, water holes can form in the extensive mud flats causing luxuriant plant growth. More than 150 species of plants and 100 species of animals were known to be exploited as foodstuffs by the Bushmen [24]. They typically gather roots, berries, fruits, melons, nuts, leafy greens and edible gums [25]. Game taken mainly consists of wildebeest, gemsbok and giraffes. They also hunt birds and reptiles, occasionally collecting honey when available. The animal food component is scarce and unpredictable, rendering meat as only of secondary dietary importance [25]. Therefore, the consumption of plant foods normally constitutes between 60 and 80% of the diet [26-28]

The Fuegian sample consists of seven specimens belonging to different tribes from Tierra del Fuego. The Fuegian Archipelago is situated in the southern extreme of South America, is divided politically between Chile and Argentina and separated from the mainland by the Straits of Magellan. The territory of Tierra del Fuego is quite mountainous with peaks surpassing elevations of 2,000 m. The climate is characterized by short summers where the average temperature is around 10°C, while the winters are long and wet with temperatures around 0°C. The northeastern areas experience strong winds and low precipitation, whereas the southwestern areas are wet, foggy and windy most of the year. Only 30% of the Fuegian Archipelago is covered by forests which are distributed along the slopes of the mountains in the southern and western part. In the north-east areas, the vegetation is scarce and mainly composed of moor. The southernmost islands are characterized by a subantarctic climate where there is no tree cover and the vegetation is primarily composed of shrubs and tundra-type vegetation.

The Fuegians subsistence was based mostly on the consumption of animal protein. The orginal tribes are considered to be functionally extinct as definable ethnic groups [29]. The Fuegians were divided into four distinct groups each with his own language and customs: the Alcaloof, the Yahgan, the Ona and the Aush [30, 31]. The Alcaloof inhabited the western part of the archipelago. The Ona occupied the forests of the northern and eastern part of the main island ending their southern range along Lake Kami. This constituted a natural barrier between them and the Yahgan tribe whose territory extended from Desolation Bay through the southern islands as far south as Cape Horn [31]. The Aush (or Eastern Ona) lived on the south-eastern tip of the main island. The diet of the Yahgan and the Alcaloof was mainly composed of fish, shellfish, crabs, birds and seals. In addition, the Yahgans were also known to collect edible berries [31]. The Ona and the Aush lived almost entirely on guanaco meat, occasionally hunting birds and exploiting stranded whales [31]. Not much information is available for the individual specimens themselves.

Fifteen specimens come from Vancouver Island and the territories situated along the Columbia River. The climate is maritime and characterized by cool summers, wet, mild winters and dominated by temperate rainforest [32]. In the southern part, along the costal lowland of Washington and Oregon State, the vegetation becomes more open with the formation of grasslands and prairies [32]. The subsistence of these populations was mainly based on the exploitation of animal proteins. The coastal plains were rich in neritic fauna including a great variety of fish, shellfish (crustaceans, molluscs), birds and sea mammals. In addition, beached dolphins and whales were exploited when possible. The molluscs were steamed and often smoked and preserved [32]. However, their subsistence economy was based mostly on the exploitation of salmon which were abundant and available at predictable times and places [32]. They were consumed fresh with the remainder dried, smoked and stored. Terrestrial mammals such as deer, elk and wild goat were also hunted. Plant foods were more important in the southern areas than in the north. Berries, ferns, bracken, acorns and hazelnuts were also collected when available were to supplement the winter diet [32]. Roots and corns were also made into flour.

The Inuit sample is composed of ten specimens with a rather heterogeneous distribution and includes individuals from the western coast of Greenland, the northern islands of the Nunavut region (Canada) and the Seward Peninsula of western Alaska These regions are characterized by a maritime arctic climate with short, mild summers (mean temperatures ~10°C), and cold, long, stormy winters. Plant growth is limited by permafrost and so the ecozone is dominated by low tundra mosses and lichens. The Inuit diet is almost entirely composed of animal protein. The Inuit living north of Hudson Bay and west of Baffin Bay, mainly base their winter diet on seals, eating all parts of the animal including the blood [33]. In spring, they subsist primarily on walrus meat while occasionally hunting polar bears and musk ox. In summer, they rely more on terrestrial animals especially caribou but also include fish (especially salmon), birds (ducks and geese) and vegetable materials such as berries, roots and some algae although the consumption of plant foods is not large [33]. The Ipiutak, from the Alaskan west coast, have similar diet hunting sea mammals (seals and walrus) in addition to caribou [34, 35]. Inuit from the Greenland west coast primarily exploit aquatic animal food sources due to the disappearance of caribou and musk ox [36]. They subsist mainly on seal meat but also hunt other animals such as walrus, narwhal, beluga, polar bear and arctic hare [36]. In addition, they exploit birds and their eggs which are seasonally numerous and easily accessible [36, 37]. The Inuit often eat their food raw but sometimes frozen or dried [33]. De Poncis [38] and Balikci [39] reported the chewing of seal skin for prolonged periods of time.

The Aborigines inhabited the entire Australian continent under various geographic and climatic conditions ranging from the northern tropical coastal regions through the central deserts to the cool, temperate southern regions [40]. They were omnivorous but generally able to secure large quantities of animal protein [41]. The diet composition and the relative proportion of plant and animal foods were greatly influenced by seasonal changes and geographic location [40]. Animal protein was composed of mammals, reptiles, birds, shellfish and insects. Almost every edible part of the animal was consumed including muscles, fat deposits and internal organs [40]. They exploited a variety of plant foods including tuberous roots, seeds, fruits, berries, nuts, beans and honey. Food was generally cooked by roasting on coals or baking in an earthen oven [40]. Sometimes it was dried and stored. Many plant foods were simply eaten fresh [40].

**References**

1. Rink WJ, Schwarcz HP, Smith FH, Radovčić J (1995) ESR ages for Krapina hominids. Nature 378: 24.

2. Van Andel TH, Tzedakis PC (1996) Paleolithic landscapes of Europe and environs, 150,000-25,000 years ago: an overview. Quaternary Sci Rev 15: 481-500.

3. Valladas H, Mercier N, Hovers E, Frojet L, Joron JL, et al. (1999) TL dates for the Neandertal site of Amud Cave, Israel. J Archaeol Sci 26: 259-268.

4. Grün R, Stringer C (2000) Tabun revisited: revised ESR chronology and new ESR and U-series analyses of dental material from Tabun C1. J Hum Evol 39: 601-612.

5. Hallin KA, Schoeninger MJ, Schwarcz HP, (2002) Evidence for summer rains during Neandertal occupation at Amud, Israel: the stable isotope data. Am J Phys Anthropol 117, S34: 81.

6. Hallin KA, Schoeninger MJ,Schwarcz HP (2008) Paleoclimate: Neandertal and early modern human occupation at Amud and Qafzeh, Israel. PalaeoAntrhopology 2008, Annual Meeting Abstracts: A11.

7. Cowgill LW, Trinkaus E, Zeder MA (2007) Shanidar 10: A Middle Paleolithic immature distal lower limb from Shanidar Cave, Iraqi Kurdistan. J Hum Evol 53: 213-223.

8. Trinkaus E (1983) The Shanidar Neandertals. New York: Academic Press.

9. Schwarcz HP, Grün R, Vandermeersch B, Bar-Yosef O, Valladas H, et al. (1988) ESR dates for the hominid burial site of Qafzeh in Israel. J Hum Evol 17: 733-737.

10. Oakley KP, Campbell BG, Molleson TI (1971) Catalogue of Fossil Hominids. Part II: Europe. London: Trustees of the British Museum, Natural History.

11. Quintard A, Maureille B, Tillier A-M, Turq A, Morala A (2007) New Middle Palaeolithic human remains from Las Pelenos (Monsempron-Libos, France). PalaeoAnthropology 2007, Annual Meeting Abstracts: A26.

12. Mellars P, Grün R (1991) Comparison of the electron spin resonance and thermoluminescence dating methods: results of ESR dating at Le Moustier (France). Cambridge Archaeol J 1: 269-276.

13. Valladas H, Geneste JM, Joron JL, Chadelle JP (1986) Thermo-luminescence dating of Le Moustier (Dordogne, France). Nature 322: 452-454.

14. Krings M, Capelli C, Tschentscher F, Geisert H, Meyer S, et al. (2000) A view of Neandertal genetic diversity. Nature Genetics 26: 144-146.

15. Wild EM, Teschler-Nicola M, Kutschera W, Steier P, Trinkaus T, et al. (2005) Direct dating of Early Upper Palaeolithic human remains from Mladeč. Nature 435: 332-335.

16. Pacher M (2006) Large mammal remains from the Mladeč Caves and their contribution to site formation processes. In: Teschler-Nicola M, editor. Early modern humans at the Moravian gate. Vienna: Springer. pp. 99-148.

17. Kuzmin YV (2004) AMS 14C age of the Upper Palaeolithic skeletons from Sungir site, Central Russian Plain. Nucl Instrum Meth B 223/224: 731-734.

18. Buzhilova AP (2005) The environment and health condition of the Upper Palaeolithic Sunghir people of Russia. JPhys Anthropol Appl Hum Sci 24: 413-418.

19. Formicola V, Pettitt PB, Del Lucchese A (2004) A Direct AMS Radiocarbon date on the Barma Grande 6 Upper Paleolithic skeleton. Curr Anthropol 45: 114-118.

20. Follieri M, Giardini M, Magri D, Sadori L (1998) Palynostratigraphy of the last glacial period in the volcanic region of Central Italy. Quatern Int 47/48: 3-20.

21. Mellars PA (1987) Radiocarbon accelerator dating of French Upper Palaeolithic sites. Curr Anthropol 28: 128-133.

22. Théry-Parisot I (2002) Fuel management (bone and wood) during the Lower Aurignacian in the Pataud rock shelter (Lower Palaeolithic, Les Eyzies de Tayac, Dordogne, France). Contribution of Experimentation. J Archaeol Sci 29: 1415-1421.

23. Pacher H-M (1961) Anthropologische Untersuchungen an den Skeletten der Rudolf Pöch'schen Buschmannsammlung. Graz: H. Bölhaus.

24. Sahlins MD (1968) Notes on the original affluent society. In: Lee RB, De Vore I, editors. Man the hunter. Chicago: Aldine Publishing Company. pp. 85-89.

25. Lee RB (1978) Ecology of a contemporary San people. In: Tobias P, editor. The Bushmen: San hunters and herders of Southern Africa, Cape Town: Human & Rousseau. pp. 98-114.

26. Lee RB (1973) Mongongo: Ethnography of a major wild food resource. Ecol Food Nutr 2: 307-321.

27. Lee RB, DeVore I (1976) Kalahari hunter-gatherers: Studies of the !Kung San and their neighbors. Cambridge: Harvard University Press.

28. Silberbauer G (1981) Hunter and habitat in the central Kalahari desert. Cambridge: Cambridge University Press.

29. García-Bour J, Pérez-Pérez A, Álvarez S, Fernández E, López-Parra A-M, et al. (2003) Early population differentiation in extinct aborigines from Tierra del Fuego-Patagonia: Ancient mtDNA sequences and Y-Chromosome STR characterization. Am J Phys Anthropol 123: 361-370.

30. Furlong CW (1917) Some Effects of Environment on the Fuegian Tribes. Geogr Rev 3: 1-15.

31. Bridges EL (1948) Uttermost part of the Earth. A history of Tierra del Fuego and the Fuegians. London: Hodder & Stoughton.

32. Ames KM, Maschner DG (1999) Peoples of the Northwest Coast. Their archaeology and prehistory. London: Thames & Hudson.

33. Sinclair HM (1953) The diet of Canadian Indians and Eskimos. Proc Nutr Soc 12: 69-82.

34. Lester CW, Shapiro HL (1968) Vertebral arch defects in the lumbar vertebrae of prehistoric American Eskimos. Am J Phys Anthropol 28: 43-48.

35. El Zaatari S (2007) Ecogeographic variation in Neanderthal dietary habits: evidence from microwear texture analysis. Ph.D. Dissertation. New York: Stony Brook University.

36. Heinbecker P (1928) Studies on the metabolism of Eskimos. J Biol Chem 80: 461-475.

37. Gotfredsen AB (1997) Sea Bird Exploitation on Coastal Inuit Sites, West and Southeast Greenland. Int J Osteoarchaeol 7: 271-286.

38. DePoncins G (1941) Kabloona. New York: Reynal & Hitchcock.

39. Balikci A (1970) The Netsilik Eskimo. New York: Natural History Press.

40. O‘Dea K, Jewell PA, Whiten A, Altmann SA, Strickland SS, et al. (1991) Traditional diet and food preferences of Australian aboriginal hunter-gatherers. Phyl Trans Biol Sci 334: 233-241.

41. Brand-Miller JC, Holt SHA (1998) Australian aboriginal plant foods: a consideration of their nutritional composition and health implications. Nut Res Rev 11: 5-23.
